# Supplementary material for: DASH Dietary Pattern and Cardiometabolic Outcomes: An Umbrella Review of Systematic Reviews and Meta-Analyses
Source: Nutrients. 2019 Feb 5;11(2):338. doi: 10.3390/nu11020338 (PMC6413235; doi:10.3390/nu11020338)
Supplement: Supplementary file 1 [file nutrients-11-00338-s001.pdf]

nutrients

MDPI

DASH dietary pattern and cardiometabolic health: An umbrella review of systematic reviews and meta-analyses

Supplemental Tables & Figures

Table S1: Search strategy for the identification of systematic reviews and meta-analyses assessing the relationships of the DASH dietary pattern with incident cardiometabolic diseases and on cardiometabolic risk factors 3

Table S2: Characteristics of prospective cohort studies investigating the DASH dietary pattern and CVD incidence 4

Table S3: Analysis of confounding variables among studies of the DASH dietary pattern and CVD incidence 7

Table S4: Newcastle-Ottawa Scale (NOS) for Assessing the Quality of Cohort Studies for Studies of the DASH diet and CVD incidence 10

Table S5: GRADE assessments of systematic reviews and meta-analyses of prospective cohort studies assessing the relationship between consumption of the DASH dietary pattern and cardiometabolic disease incidence 11

Table S6: Characteristics of prospective cohort studies investigating the DASH dietary pattern and incidence of CHD 12

Table S7: Analysis of confounding variables among studies of the DASH dietary pattern and incidence of CHD 14

Table S8: Newcastle-Ottawa Scale (NOS) for Assessing the Quality of Cohort Studies for Studies of the DASH diet and incidence of CHD 16

Table S9: Characteristics of prospective cohort studies investigating the DASH dietary pattern and incidence of stroke 17

Table S10: Analysis of confounding variables among studies of the DASH dietary pattern and incidence of stroke 19

Table S11: Newcastle-Ottawa Scale (NOS) for Assessing the Quality of Cohort Studies for Studies of the DASH diet and incidence of stroke 21

Table S12: Characteristics of prospective cohort studies investigating the DASH dietary pattern and incidence of diabetes 22

Table S13: Analysis of confounding variables among studies of the DASH dietary pattern and incidence of diabetes 24

Table S14: Newcastle-Ottawa Scale (NOS) for Assessing the Quality of Cohort Studies for Studies of the DASH diet and incidence of diabetes 26

Table S15: Characteristics of controlled trials investigating the DASH dietary pattern and blood pressure 27

Table S16: GRADE assessment of the systematic review and meta-analysis of controlled trials assessing the effect of the DASH dietary pattern on cardiometabolic risk factors 31

Table S17: Characteristics of controlled trials investigating the DASH dietary pattern and Total-C and LDL-C 33

Table S18: Characteristics of controlled trials investigating the DASH dietary pattern and HDL-C 36

Table S19: Characteristics of controlled trials investigating the DASH dietary pattern and triglycerides 39

Table S20: Search strategy for controlled clinical trials assessing the effect of the DASH diet intervention on HbA1c 42
Table S21: Characteristics of controlled trials investigating the DASH dietary pattern and HbA1c 44
Table S22: Characteristics of controlled trials investigating the DASH dietary pattern and blood glucose 45
Table S23: Characteristics of controlled trials investigating the DASH dietary pattern and fasting insulin 47
Table S24: Characteristics of controlled trials investigating the DASH dietary pattern and HOMA-IR 50
Table S25: Characteristics of controlled trials investigating the DASH dietary pattern and body weight 52
Table S26: Characteristics of controlled trials investigating the DASH dietary pattern and CRP 55
Figure S1: Risk of Bias summary for all controlled trials included in the systematic reviews and meta-analyses of cardiovascular risk factors 58
Figure S2: Risk of Bias graph for all controlled trials included in the systematic reviews and meta-analyses of cardiovascular risk factors 59
Figure S3: Literature search for the effect of the DASH diet intervention on HbA1c in controlled clinical trials 60
Figure S4: Forest plot for controlled trials investigating the effect of the DASH dietary pattern on HbA1c. 61

Table S1: Search strategy for the identification of systematic reviews and meta-analyses assessing the relationships of the DASH dietary pattern with incident cardiometabolic diseases and on cardiometabolic risk factors

**MEDLINE**	**Embase**
1. dietary approaches to stop hypertension.mp	1. dietary approaches to stop hypertension.mp
2. DASH.mp	2. DASH.mp
3. 1 or 2	3. 1 or 2
4. meta-analysis.mp	4. meta-analysis.mp
5. 3 AND 4	5. 3 AND 4

For all databases, the original search date was October 1, 2017 and was updated on January 3, 2019.

Table S2: Characteristics of prospective cohort studies investigating the DASH dietary pattern and CVD incidence

Study, yr	Cohort	Country	No. of participants	Outcome	No. of incident cases	Age, yr	Duration of study, yr	Dietary intake assessment (at baseline)	DASH exposure assessment*	Method of outcome assessment	Funding Source ‡	Study Quality**
**Agnoli et al. 2011**	EPICOR	Italy	44544	Stroke incidence	178	M:35-64; F:35-74	7.89	validated semi quantitative FFQ	DASH score out of 40, Tertile 1 vs 3.	Record linkage	Agency & Industry	High
**Bertoia et al. 2013**	WHI	United States	93122	Sudden cardiac death	237	F: 50-79	10.5	validated semi quantitative FFQ	DASH score out of 40, Quintile 1 vs 5.	Record linkage	Agency	High
**Fitzgerald et al. 2011**	WHS	United States	34827	CVD incidence	1094	F: ≥45	14.6	semi quantitative FFQ	DASH score out of 40, Quintile 1 vs 5.	Record linkage	Agency	High
**Folsom et al. 2007**	IWHS	United States	20993	CVD mortality	1121	F: 55-69	≤16	validated semi quantitative FFQ	DASH score out of 11, Quintile 1 vs 5	Record linkage	Agency	Low
**Fung et al. 2008 (1)**	NHS	United States	88517	CHD incidence	3105	F: 30-55	24	validated semi quantitative FFQ	DASH score out of 40, Quintile 1 vs 5.	Record linkage	Agency	High
**Fung et al. 2008 (2)**	NHS	United States	88517	Stroke incidence	2317	F: 30-55	24	validated semi quantitative FFQ	DASH score out of 40, Quintile 1 vs 5.	Record linkage	Agency	High

**Levitan et al. 2009 (M)**	Cohort of Swedish Men	Sweden	38987	CHD incidence	807	M: 45-79	9	validated semi quantitative FFQ	DASH score out of 40, Quartile 1 vs 4.	Record linkage	Agency	High
**Levitan et al. 2009 (F)**	Swedish Mammography Cohort	Sweden	36019	CHD incidence	443	F: 48-83	7	validated semi quantitative FFQ	DASH score out of 40, Quartile 1 vs 4.	Record linkage	Agency	High
**Lin et al. 2013**	CVDFACTS	China	2061	Stroke incidence	123	≥20	≤12	validated semi quantitative FFQ	DASH food score & DASH nutrient score, Tertile 1 vs 3	Record linkage	Agency	High
**Reedy et al. 2014 (M)**	NIH-AARP Diet & Health Study	United States	242321	CVD mortality	15497	M: 50-71	15	validated semi quantitative FFQ	DASH score out of 40, Quintile 1 vs. 5	Record linkage	NR	High
**Reedy et al. 2014 (F)**	NIH-AARP Diet & Health Study	United States	182341	CVD mortality	8005	F: 50-71	15	validated semi quantitative FFQ	DASH score out of 40, Quintile 1 vs. 5	Record linkage	NR	High

* What was compared in the cohort, e.g. DASH score out of 10, Quintile 1 vs. 10

** A total score of 6 or greater was considered high-quality and a total score of 5 or smaller was considered low-quality

‡ Agency funding is that from government, university or not-for-profit health agency sources.

CVDFACTS, CardioVascular Disease risk FACtor Two-township Study; DASH, dietary approaches to stop hypertension; IWHS, Iowa Women's Health Study; EPICOR, long-tErm follow uP of antithrombotic management patterns In acute CORonary syndrome patients; F, female; M, male;

NHS, Nurses' Health Study; NIH-AARP, National Institutes of Health-American Association of Retired Persons; NR, not reported; WHI, Women's Health Initiative; WHS, Women's Health Study; yr, year

Table S3: Analysis of confounding variables among studies of the DASH dietary pattern and CVD incidence

**Study**	**Agnoli et al. 2011**	**Bertoia et al. 2013**	**Fitzgerald et al. 2011**	**Folsom et al. 2007**	**Fung et al. 2008 (1-CHD)**	**Fung et al. 2008 (2-stroke)**	**Levitan et al. 2009 (M)**	**Levitan et al. 2009 (F)**	**Lin et al. 2013**	**Reedy et al. 2014 (M)**	**Reedy et al. 2014 (F)**
**Number of variables in fully adjusted model**	7	12	14	11	14	14	12	13	9	11	12
**Number of multivariable models presented**	2	3	10	2	3	3	2	2	1	1	1
**Timing of measurement of confounding variables**	Baseline	Baseline	Baseline	Baseline	Baseline	Baseline	Baseline	Baseline	Baseline	Baseline	Baseline
**Pre-specified primary confounding variable***											
Age	x	x	x	x	x	x	x	x	x	x	x
**Pre-specified secondary confounding variables***											
Markers of overweight/obesity (Body mass index, weight, waist circumference, waist to hip ratio)	x	x	x	x	x	x	x	x	x	x	x
Family history of CVD			x		x	x	x	x			
Presence of diabetes		x					x	x		x	x
Energy Intake	x	x	x	x	x	x	x	x		x	x
Physical activity								x			
Total physical activity		x	x	x	x	x	x		x	x	x
Sex	x		x	x	x	x	x	x	x	x	x
Smoking status	x	x	x	x	x	x	x	x	x	x	x
Hypertension (or		x	x				x		x		

meds)											
Dyslipidemia (or meds)			x				x				
**Other confounding variables**											
Smoking pack-years			x	x							
Marital status							x			x	x
Living alone								x			
Alcohol consumption			x	x	x	x			x	x	x
Multivitamin use				x	x	x					
Omega-3 use					x	x					
Trans fat					x	x					
Aspirin use					x	x					
Income		x									
Urinary sodium/creatinine									x		
Pulse		x									
**Biomarkers**											
Blood pressure									x		
High cholesterol								x			
**Medical history**											
History of CHD		x									
History of cardiac failure		x									
History of hypertension								x			
History of infarction							x	x			
Menopausal status			x		x	x					
Post-menopausal hormone use			x	x	x	x		x			x
**Socio-economic factors**											
Educational	x		x	x			x	x		x	x

attainment											
**Ethno-cultural/geographical factors**											
Country of birth/Ethnicity		x								x	x
Participant center	x										
**Others**											
Randomization status			x								

*These are used in the assessment of the comparability category in the Newcastle-Ottawa Score

CHD, coronary heart disease; CVD, cardiovascular disease; DASH, dietary approaches to stop hypertension; F, female; M, male; meds, medication use

Table S4: Newcastle-Ottawa Scale (NOS) for Assessing the Quality of Cohort Studies for Studies of the DASH diet and CVD incidence

Study, yr	Selection*	Outcome†	Comparability‡	Total§	Study Quality**
Agnoli et al. 2011	2	3	1	6	High
Bertoia et al. 2013	3	3	2	8	High
Fitzgerald et al. 2011	2	3	2	7	High
Folsom et al. 2007	2	2	1	5	Low
Fung et al. 2008 (1)	2	3	2	7	High
Fung et al. 2008 (2)	2	3	2	7	High
Levitan et al. 2009 (M)	2	3	2	7	High
Levitan et al. 2009 (F)	2	3	2	7	High
Lin et al. 2013	3	3	1	7	High
Reedy et al. 2014 (M)	2	3	1	6	High
Reedy et al. 2014 (F)	2	3	1	6	High

*Maximum 4 points awarded for cohort representativeness, selection of non-exposed cohort, exposure assessment, and demonstration outcome not present at baseline

†Maximum 3 points awarded for follow-up length, adequacy of follow-up, and outcome assessment

‡ Maximum 2 points awarded for controlling for the pre-specified primary confounding variable (age) and 5 of the 7 secondary (markers of overweight/obesity, family history of CVD, energy intake, physical activity, sex, smoking, hypertension, dyslipidemia) confounding variables (refer to Supplemental Table S3)

§ A maximum of 9 points could be awarded

** A total score of 6 or greater was considered high-quality and a total score of 5 or smaller was considered low-quality

DASH, dietary approaches to stop hypertension; F, female; M, male; yr, year

Table S5: GRADE assessments of systematic reviews and meta-analyses of prospective cohort studies assessing the relationship between consumption of the DASH dietary pattern and cardiometabolic disease incidence

Cardiometabolic Disease Risk	No. of studies	Design	Risk of bias[1]	Inconsistency	Indirectness	Imprecision	Other considerations	Quality
**CVD Incidence**								
Schwingshackl et al. 2015	11	observational studies	no serious risk of bias	no serious inconsistency	no serious indirectness	no serious imprecision	none[2]	⊕⊕OO LOW
**CHD Incidence**								
Salehi-Abargouei et al. 2013	3	observational studies	no serious risk of bias	no serious inconsistency	serious[3]	no serious imprecision	none[4]	⊕OOO VERY LOW
**Stroke Incidence**								
Salehi-Abargouei et al. 2013	3	observational studies	no serious risk of bias	no serious inconsistency	no serious indirectness	no serious imprecision	none[4]	⊕⊕OO LOW
**Diabetes Incidence**								
Jannasch et al. 2017	5	observational studies	no serious risk of bias	serious[5]	no serious indirectness[6]	no serious imprecision	none[7]	⊕OOO VERY LOW

[1] Newcastle Ottawa quality assessment Scale was used to assess study quality across the following domains: selection (4 points), comparability (2 points), and outcome (3 points). A total score of 6 or greater was considered high-quality and a total score of 5 or smaller was considered low-quality. If the majority of included studies were of high study quality, there is no serious risk of bias

[2] Slight asymmetry was detected in the funnel plot, however, results from the Egger's test showed no evidence of publication bias

[3] Findings are not generalizable given that the three prospective cohort studies were conducted in middle-aged or elderly women

[4] Asymmetry in the funnel plots from the Begg's test showed evidence of publication bias, however, there was no evidence of publication bias using Egger's test. Additionally, there were <10 studies which may have not been enough power to distinguish chance from real funnel plot asymmetry

[5] Serious inconsistency due to high heterogeneity (I^2 = 62%; P=0.03), and no subgroup analyses were performed to attempt to explain heterogeneity

[6]Although 4 of the 5 cohorts were conducted in the united states, we did not downgrade for indirectness since the united states has multiethnic population and the European cohort included 8 countries.

[7] There was no asymmetry in the funnel plot and the results from the Egger's and Begg's tests did not show evidence of publication bias, however, there were <10 studies which may have not been enough power to distinguish chance from real funnel plot

Table S6: Characteristics of prospective cohort studies investigating the DASH dietary pattern and incidence of CHD

Study, yr	Cohort	Country	No. of participants	Outcome	No. of incident cases	Age, yr	Duration of study, yr	Dietary intake assessment (at baseline)	DASH exposure assessment*	Method of outcome assessment	Funding Source ‡	Study Quality**
**Fitzgerald et al. 2011**	WHS	United States	34827	CHD morbidity & mortality	430	F: ≥45	14.6	semi quantitative FFQ	DASH score out of 38, Quintile 1 vs 5.	Record linkage	Agency	High
**Folsom et al. 2007**	IWHS	United States	20993	CHD mortality	620	F: 55-69	≤16	validated semi quantitative FFQ	DASH score out of 11, Quintile 1 vs 5	Record linkage	Agency	Low
**Fung et al. 2008 (1)*** **	NHS	United States	88517	Total CHD	3105	F: 30-55	24	validated semi quantitative FFQ	DASH score out of 40, Quintile 1 vs 5.	Record linkage	Agency	High
**Fung et al. 2008 (2)*** **	see above			CHD morbidity	2129							
**Fung et al. 2008 (3)*** **	see above			CHD mortality	976							

* What was compared in the cohort, e.g. DASH score out of 10, Quintile 1 vs. 10

** A total score of 6 or greater was considered high-quality and a total score of 5 or smaller was considered low-quality

*** All 3 outcomes were combined as one (fatal and nonfatal CHD) in the analysis of Salehi-Abargouei et al. 2013

‡ Agency funding is that from government, university or not-for-profit health agency sources.

CHD, coronary heart disease; DASH, dietary approaches to stop hypertension; F, female; FFQ, food frequency questionnaire; IWHS, Iowa Women's Health Study; NHS, Nurses' Health Study; WHS, Women's Health Study; yr, year

Table S7: Analysis of confounding variables among studies of the DASH dietary pattern and incidence of CHD

**Study**	**Fitzgerald et al. 2011**	**Folsom et al. 2007**	**Fung et al. 2008**
**Number of variables in fully adjusted model**	15	11	14
**Number of multivariable models presented**	8	2	3
**Timing of measurement of confounding variables**	baseline	baseline	baseline and every 2 years
**Pre-specified primary confounding variable***			
Age	x	x	x
**Pre-specified secondary confounding variables***			
Markers of overweight/obesity (Body mass index, weight, waist circumference, waist to hip ratio)	x	x	x
Family history of CVD	x		x
Presence of diabetes			
Energy Intake	x	x	x
Physical activity			
Total physical activity	x	x	x
Sex	x	x	x
Smoking status	x	x	x
Hypertension (or meds)	x		
Dyslipidemia (or meds)	x		
**Other confounding variables**			
Smoking pack-years	x	x	
Alcohol consumption	x	x	x
Multivitamin use		x	x
Omega-3 use			x
Trans fat			x
Aspirin use			x

**Medical history**			
Menopausal status	x		x
Post-menopausal hormone use	x	x	x
**Socio-economic factors**			
Educational attainment	x	x	
**Others**			
Randomization status	x		

*These are used in the assessment of the comparability category in the Newcastle-Ottawa Score

CHD, coronary heart disease; CVD, cardiovascular disease; DASH, dietary approaches to stop hypertension; meds, medication use

Table S8: Newcastle-Ottawa Scale (NOS) for Assessing the Quality of Cohort Studies for Studies of the DASH diet and incidence of CHD

Study, yr	Selection*	Outcome†	Comparability‡	Total§
**Fitzgerald et al. 2011**	**2**	**3**	**2**	**7**
**Folsom et al. 2007**	**2**	**2**	**1**	**5**
**Fung et al. 2008**	**2**	**3**	**2**	**7**

*Maximum 4 points awarded for cohort representativeness, selection of non-exposed cohort, exposure assessment, and demonstration outcome not present at baseline

†Maximum 3 points awarded for follow-up length, adequacy of follow-up, and outcome assessment

‡ Maximum 2 points awarded for controlling for the pre-specified primary confounding variable (age) and 5 of the 7 secondary (markers of overweight/obesity, family history of CVD, energy intake, physical activity, sex, smoking, hypertension, dyslipidemia) confounding variables (refer to Supplemental Table S7)

§ A maximum of 9 points could be awarded

** A total score of 6 or greater was considered high-quality and a total score of 5 or smaller was considered low-quality

CHD, coronary heart disease; DASH, dietary approaches to stop hypertension; yr, year

Table S9: Characteristics of prospective cohort studies investigating the DASH dietary pattern and incidence of stroke

Study, yr	Cohort	Country	No. of participants	Outcome	No. of incident cases	Age, yr	Duration of study, yr	Dietary intake assessment (at baseline)	DASH exposure assessment*	Method of outcome assessment	Funding Source‡	Study Quality**
**Agnoli et al. 2011**	EPICOR	Italy	40681	Stroke incidence	178	M:35-64; F:35-74	7.89	validated semi quantitative FFQ	DASH score out of 40, Tertile 1 vs 3.	Record linkage	Agency, Industry	High
**Folsom et al. 2007**	IWHS	United States	20993	Stroke mortality	236	F: 55-69	≤16	validated semi quantitative FFQ	DASH score out of 11, Quintile 1 vs 5	Record linkage	Agency	Low
**Fung et al. 2008 (1)*** **	NHS	United States	88517	Total Stroke	2317	F: 30-55	24	validated semi quantitative FFQ	DASH score out of 40, Quintile 1 vs 5.	Record linkage	Agency	High
**Fung et al. 2008 (2)*** **	see above			Stroke morbidity	1242							
**Fung et al. 2008 (3)*** **	see above			Stroke mortality	440							

* What was compared in the cohort, e.g. DASH score out of 10, Quintile 1 vs. 10

** A total score of 6 or greater was considered high-quality and a total score of 5 or smaller was considered low-quality

*** All 3 outcomes were combined as one (fatal and nonfatal CHD) in the analysis of Salehi-Abargouei et al. 2013

‡ Agency funding is that from government, university or not-for-profit health agency sources

DASH, dietary approaches to stop hypertension; EPICOR, long-tErm follow uP of antithrombotic management patterns In acute CORonary syndrome patients; F, female; FFQ, food frequency questionnaire; IWHS, Iowa Women's Health Study; NHS, Nurses' Health Study; yr, year

Table S10: Analysis of confounding variables among studies of the DASH dietary pattern and incidence of stroke

**Study**	**Agnoli et al. 2011**	**Folsom et al. 2007**	**Fung et al. 2008**
**Number of variables in fully adjusted model**	7	11	14
**Number of multivariable models presented**	2	2	3
**Timing of measurement of confounding variables**	baseline	baseline	baseline and every 2 years
**Pre-specified primary confounding variable***			
Age	x	x	x
**Pre-specified secondary confounding variables***			
Markers of overweight/obesity (Body mass index, weight, waist circumference, waist to hip ratio)	x	x	x
Family history of CVD			x
Presence of diabetes			
Energy Intake	x	x	x
Physical activity			
Total physical activity		x	x
Sex	x	x	x
Smoking status	x	x	x
Hypertension (or meds)			
Dyslipidemia (or meds)			
**Other confounding variables**			
Smoking pack-years		x	
Alcohol consumption		x	x
Multivitamin use		x	x
Omega-3 use			x
Trans fat			x
Aspirin use			x
**Medical history**			

Menopausal status			x
Post-menopausal hormone use		x	x
**Socio-economic factors**			
Educational attainment	x	x	
**Ethno-cultural/geographical factors**			
Participant center	x		

*These are used in the assessment of the comparability category in the Newcastle-Ottawa Score

CVD, cardiovascular disease; DASH, dietary approaches to stop hypertension; meds, medication use

Table S11: Newcastle-Ottawa Scale (NOS) for Assessing the Quality of Cohort Studies for Studies of the DASH diet and incidence of stroke

Study, yr	Selection*	Outcome†	Comparability‡	Total§	Study Quality**
**Agnoli et al. 2011**	3	3	1	7	High
**Folsom et al. 2007**	2	2	1	5	Low
**Fung et al. 2008**	2	3	2	7	High

*Maximum 4 points awarded for cohort representativeness, selection of non-exposed cohort, exposure assessment, and demonstration outcome not present at baseline

†Maximum 3 points awarded for follow-up length, adequacy of follow-up, and outcome assessment

‡ Maximum 2 points awarded for controlling for the pre-specified primary confounding variable (age) and 5 of the 7 secondary (markers of overweight/obesity, family history of CVD, energy intake, physical activity, sex, smoking, hypertension, dyslipidemia) confounding variables (refer to Supplemental Table S10)

§ A maximum of 9 points could be awarded

** A total score of 6 or greater was considered high-quality and a total score of 5 or smaller was considered low-quality

DASH, dietary approaches to stop hypertension; yr, year

Table S12: Characteristics of prospective cohort studies investigating the DASH dietary pattern and incidence of diabetes

Study, yr	Cohort	Country	No. of participants	No. of incident cases	Age, yr	Duration of study, yr	Dietary intake assessment (at baseline)	DASH diet exposure assessment*	Method of outcome assessment	Funding Source ‡	Study Quality**
**De Koning et al. 2011**	HPFS	United States	41615	2795	40-75	≤ 20	FFQ	DASH score out of 40, Quintile 1 vs 5.	Self-reported with validation by medical record review	Agency	High
**de Oliveira Otto et al. 2015**	MESA	United States	5160	588	45-84	10	FFQ	DASH score out of 40, Quintile 1 vs 5.	Independent Assessment	Agency, Industry	High
**Jacobs et al. 2015**	Multiethnic Cohort	United States	89195	11217	45-75	11 to 14	FFQ	DASH score out of 40, Quintile 1 vs 5.	Self-Reported & Record Linkage	Agency	High
**EPIC InterAct Consortium Kröger, J. et al. 2014**	EPIC-InterAct	Europe (included 8 countries)	21616	8883	25-79	7 to 16	FFQ	DASH score out of 10, Quintile 1 vs 5.	Self-report, linkage to primary-care registers, secondary-care registers, medication use (drug	Agency	Low

									registers), hospital admissions and mortality data		
**Liese et al. 2009**	IRAS	United States	822	129	40-69	5	FFQ	DASH score out of 80, Tertile 1 vs 3	OGTT or hypoglycemic medication use	Agency	High

* What was compared in the cohort, e.g. DASH score out of 10, Quintile 1 vs. 10

** A total score of 6 or greater was considered high-quality and a total score of 5 or smaller was considered low-quality

‡ Agency funding is that from government, university or not-for-profit health agency sources.

DASH, dietary approaches to stop hypertension; EPIC, European Prospective Investigation into Cancer and Nutrition study; FFQ, food frequency questionnaire; HPFS, Health Professionals Follow-up Study; OGTT, Oral glucose tolerance test; IRAS, Insulin Resistance Atherosclerosis Study; MESA, Multi-Ethnic Study of Atherosclerosis; No., number; yr, year

Table S13: Analysis of confounding variables among studies of the DASH dietary pattern and incidence of diabetes

**Study**	**De Koning et al. 2011**	**de Oliveira Otto et al. 2015**	**Jacobs et al. 2015**	**EPIC InterAct Consortium Kröger, J. et al. 2014**	**Liese et al. 2009**
**Number of variables in fully adjusted model**	6	12	8	9	13
**Number of multivariable models presented**	3	2	1	4	3
**Timing of measurement of confounding variables**	Baseline or more recently completed questionnaire	Baseline	Baseline	Baseline	Baseline
**Pre-specified primary confounding variable***					
Age		x	x	x	x
**Pre-specified secondary confounding variables***					
Markers of overweight/obesity (Body mass index, weight, waist circumference, waist to hip ratio)	x	x	x	x	x
Family history of diabetes	x				x
Energy Intake	x	x	x	x	x
Physical activity					
Total physical activity	x		x	x	x
Metabolic Equivalents (METs)		x			
Sex	x	x	x	x	x
Smoking	x	x	x	x	x
**Other confounding variables**					
Alcohol consumption		x			
Supplement use		x			

**Biomarkers**					
Glucose Tolerance Status					x
Insulin Sensitivity					x
Insulin Secretion					x
**Socio-economic factors**					
Educational attainment		x	x	x	x
**Ethno-cultural/geographical factors**					
Participant center		x		x	x
Ethnicity		x	x		x
**Others**					
Coffee intake	x				

*These are used in the assessment of the comparability category in the Newcastle-Ottawa Score

EPIC, European Prospective Investigation into Cancer and Nutrition study

Table S14: Newcastle-Ottawa Scale (NOS) for Assessing the Quality of Cohort Studies for Studies of the DASH diet and incidence of diabetes

Study, yr	Selection*	Outcome†	Comparability‡	Total§	Study Quality**
**De Koning et al. 2011**	**2**	**3**	**1**	**6**	High
**de Oliveira Otto et al. 2015**	**3**	**2**	**2**	**7**	High
**Jacobs et al. 2015**	**2**	**3**	**2**	**7**	High
**EPIC InterAct Consortium Kröger, J. et al. 2014**	**0**	**2**	**2**	**4**	Low
**Liese et al. 2009**	**3**	**3**	**2**	**8**	High

*Maximum 4 points awarded for cohort representativeness, selection of non-exposed cohort, exposure assessment, and demonstration outcome not present at baseline

†Maximum 3 points awarded for follow-up length, adequacy of follow-up, and outcome assessment

‡ Maximum 2 points awarded for controlling for the pre-specified primary confounding variable (age) and 4 of the 6 secondary (markers of overweight/obesity, family history of diabetes, energy intake, physical activity, sex, smoking) confounding variables (refer to Supplemental Table S14)

§ A maximum of 9 points could be awarded

** A total score of 6 or greater was considered high-quality and a total score of 5 or smaller was considered low-quality

EPIC, European Prospective Investigation into Cancer and Nutrition study; yr, year

Table S15: Characteristics of controlled trials investigating the DASH dietary pattern and blood pressure

Study, yr	No. of participants	Sex (% F)	Design	Country	F/U, wks	Randomized	Metabolic Phenotype	Mean Age, yr (SD)	Intervention	Comparator	Feeding/Compliance	Overall ROB Category*
Appel et al. 1997	305	49	P	USA	8	Y	Hypertensive (BP<160/80-95mmHg, not on antihypertensive meds, no poorly controlled DM / dyslipidemia)	44 (11)	DASH	typical American diet	metabolic	Low
Sacks et al. 2001	412	56	P	USA	12	Y	Hypertensive (BP<160/80-95mmHg, not on antihypertensive meds, no poorly controlled DM / dyslipidemia)	48 (10)	DASH	typical American diet	metabolic	Low
Appel et al. 2003	537	61	P	USA	24	Y	OH, Prehypertensive + Hypertensive	50.2 (8.9)	behavioural intervention Plus DASH	behavioural intervention (includes goals for exercise, reducing sodium, alcohol, etc.)	dietary advice	Low
Conlin et al. 2003	55	55	P	USA	8	Y	Hypertensive	52 (9.5)	DASH	typical American diet	metabolic	Low
Lopes et al. 2003 - L	12	50	CO	USA	4	Y	OH, Lean	39 (6.9)	DASH ("DASH-CD" = DASH combination diet, high in	low antioxidant diet	dietary advice	Unclear

									antioxidants)			
Lopes et al. 2003 - OB	12	50	CO	USA	4	Y	OB	35 (6.9)	DASH ("DASH-CD" = DASH combination diet, high in antioxidants)	low antioxidant diet	dietary advice	Unclear
Nowson et al. 2004	94	40	CO	Australia	4	N	Hypertensive	55.6 (9.9)	DASH-type diet (DASH plus increased fish, nuts, legumes, decreased sodium)	control diet (typical Australian diet)	dietary advice	Unclear
Nowson et al. 2005	54	0	P	Australia	12	Y	Hypertensive, OW (BMI 25-35kg/m2)	48.0 (9.3)	DASH-type diet (DASH plus increased fish, nuts, legumes, decreased sodium)	Low fat	dietary advice	Unclear
Azadbakht et al. 2005 - M	22	0	P	Iran	24	Y	Metabolic Syndrome	~41.2 (12.3)	weight loss DASH	weight loss	dietary advice	Unclear
Azadbakht et al. 2005 - W	54	100	P	Iran	24	Y	Metabolic Syndrome	~41.2 (12.3)	weight loss DASH	weight loss	dietary advice	Unclear
Nowson et al.	95	100	P	Australia	14	Y	Hypertensive	59.2 (4.8)	DASH-type (DASH+low	Healthy diet - general guidelines	dietary advice	Unclear

2009									sodium, with lean meat)	to reduce fat, increase breads and cereals (represents a low fat diet)	plus some supplementation of foods	
Al Solaiman et al. 2010 - L	15	80	CO	USA	3	N	Lean normotensive	36.7 (7.0)	DASH (without low fat dairy)	usual diet - low in fruit and vegetable	dietary advice	Unclear
Al Solaiman et al. 2010 - OB	15	80	CO	USA	3	N	OB, Prehypertensive + Hypertensive	40.3 (6.6)	DASH (without low fat dairy)	usual diet - low in fruit and vegetable	dietary advice	Unclear
Blumenthal et al. 2010	94	67	P	USA	16	Y	OW, Hypertensive, OH	51.8 (9.4)	DASH	usual diet	dietary advice	Low
Malloy-McFall et al. 2010	20	40	P	USA	4	Y	Prehypertensive + Hypertensive	38.3 (10.5)	DASH	usual diet	dietary advice	Low
Azadbakht et al. 2011	31	58	CO	Iran	8	Y	T2DM	55.0 (6.5)	DASH	usual diet	dietary advice	Low
Edwards et al. 2011	37	51	P	USA	12	Y	Hypertensive	47.0 (9.4)	exercise + weight loss DASH	exercise only	dietary advice	Unclear
Lin et al. 2012	20	65	P	USA	2	Y	Hypertensive	44.3 (7.8)	DASH	typical American diet	metabolic	Low
Asemi et al. 2013	34	100	P	Iran	4	Y	GDM	30.1 (6.4)	DASH	usual GDM practice	dietary advice	Low

*For ROB, an assessment was performed using the Cochrane Risk of Bias tool, including the evaluation of individual domains of risk of bias (sequence generation, allocation concealment, blinding of participants/ personnel and outcome assessors, incomplete outcome data, selective

outcome reporting). Each of the 5 domains was evaluated as either low, high or unclear ROB and the overall ROB category was determined based on the most selected category.

BMI, body mass index; BP, blood pressure; CO, crossover; DASH, dietary approaches to stop hypertension; DM, diabetes; F, female; F/U, follow-up; GDM, gestational diabetes; L, lean; M, male; meds, medication; N, no; OB, obese; OH, overall healthy; OW, overweight; P, parallel; ROB, Risk of Bias; SD, standard deviation; T2DM, type 2 diabetes; W, women; wks, weeks; Y, yes; yr; year

Table S16: GRADE assessment of the systematic review and meta-analysis of controlled trials assessing the effect of the DASH dietary pattern on cardiometabolic risk factors

Cardiometabolic Risk Factor	No. of comparisons	Design	Risk of Bias*	Inconsistency	Indirectness	Imprecision	Other Considerations	Quality
SBP	19	randomised trials	no serious risk of bias	serious[1]	no serious indirectness	no serious imprecision	none	⊕⊕⊕O MOD
DBP	19	randomised trials	no serious risk of bias	serious[2]	no serious indirectness	serious[3]	none	⊕⊕OO LOW
Total-C	13	randomised trials	no serious risk of bias	serious[4]	no serious indirectness	serious[5]	none	⊕⊕OO LOW
LDL-C	13	randomised trials	no serious risk of bias	no serious inconsistency	no serious indirectness	serious[6]	none	⊕⊕⊕O MOD
HDL-C	15	randomised trials	no serious risk of bias	serious[7]	no serious indirectness	serious[8]	none	⊕⊕OO LOW
Triglycerides	14	randomised trials	no serious risk of bias	no serious inconsistency	no serious indirectness	serious[9]	serious[10]	⊕⊕OO LOW
HbA1c**	2	randomised trials	no serious risk of bias	serious[11]	serious[12]	no serious imprecision	none[13]	⊕⊕OO LOW
Blood glucose	10	randomised trials	no serious risk of bias	serious[14]	no serious indirectness	serious[15]	none	⊕⊕OO LOW
Fasting insulin	11	randomised trials	no serious risk of bias	no serious inconsistency	no serious indirectness	serious[16]	none	⊕⊕⊕O MOD
HOMA-IR	8	randomised trials	no serious risk of bias	no serious inconsistency	no serious indirectness	serious[17]	none[18]	⊕⊕⊕O MOD
Body weight	11	randomised trials	no serious risk of bias	serious[19]	no serious indirectness	no serious imprecision	none	⊕⊕⊕O MOD
CRP	6	randomised trials	no serious risk of bias	serious[20]	no serious indirectness	serious[21]	none[22]	⊕⊕OO LOW

*manually assessed by LC, SKN and EV
**manually conducted by LC and SKN

CRP, c-reactive protein; DBP, diastolic blood pressure; HDL-C, high-density lipoprotein-cholesterol; HOMA-IR, Homeostatic Model Assessment of Insulin Resistance; LDL-C, low-density lipoprotein-cholesterol; MOD, moderate; SBP, systolic blood pressure; Total-C, total-cholesterol

[1] Serious inconsistency due to high heterogeneity (I^2 = 76%; P<0.001).
[2] Serious inconsistency due to high heterogeneity (I^2 = 49%; P=0.009).
[3] The 95% CI (-3.50, -1.70mmHg) overlaps with the minimally important difference of 2 mmHg.
[4] Serious inconsistency due to high heterogeneity (I^2 = 52%; P=0.01).
[5] The 95% CI (-12.00, -3.80mg/dL) overlaps with the minimally important difference of 3.87mg/dL.
[6] The 95% CI (-7.70, -0.30mg/dL) overlaps with the minimally important difference of 3.87mg/dL.
[7] Serious inconsistency due to high heterogeneity (I^2 = 79%; P<0.001).
[8] Although the 95% CI (-2.00, 2.10mg/dL) does not include the minimally important difference of 3.87mg/dL, neither the upper or lower bound of the 95% CI are lower or higher than 3.87mg/dL, respectively.
[9] The 95% CI (-5.60, 4.70mg/dL) overlaps with the minimally important difference of 3.87mg/dL.
[10] There is evidence of potential publication bias since funnel plots generated for triglycerides revealed some asymmetry, which was confirmed by a significant Egger regression test (P=0·01).
[11] Serious inconsistency due to high heterogeneity (I^2 = 99%; P<0.001).
[12] Serious indirectness due to <5 studies available for inclusion and lack of generalizability since one study was in those with type 2 diabetes and the other in women with gestational diabetes.
[13] Given that there were <10 studies there may have not been enough power to detect asymmetry in funnel plots.
[14] Serious inconsistency due to high heterogeneity (I^2 = 59%; P=0.008).
[15] Although the 95% CI (-7.70, -0.30mg/dL) does not include the minimally important difference of 9.1mg/dL, neither the upper or lower bound of the 95% CI are lower or higher than 9.1mg/dL, respectively.
[16] Although the 95% CI (-0.22, -0.08uU/mL) does not include the minimally important difference of 0.7uU/mL, neither the upper or lower bound of the 95% CI are lower or higher than 0.7uU/mL, respectively.
[17] Although the 95% CI (-0.15, 0.05) does not include the minimally important difference of 1, neither the upper or lower bound of the 95% CI are lower or higher than 1, respectively.
[18] Given that there were <10 studies there may have not been enough power to detect asymmetry in funnel plots.
[19] Serious inconsistency due to high heterogeneity (I^2 = 71%; P<0.001).
[20] Serious inconsistency due to high heterogeneity (I^2 = 97%; P<0.001).
[21] The 95% CI (-0.98, 0.17mg/L) overlaps with the minimally important difference of 0.5mg/L.
[22] Given that there were <10 studies there may have not been enough power to detect asymmetry in funnel plots.

Table S17: Characteristics of controlled trials investigating the DASH dietary pattern and Total-C and LDL-C

Study, yr	No. of participants	Sex (% F)	Design	Country	F/U, wks	Randomized	Metabolic Phenotype	Mean Age, yr (SD)	Intervention	Comparator	Feeding/ Compliance	Overall ROB Category*
Appel et al. 2003	537	61	P	USA	24	Y	OH, Prehypertensive + Hypertensive	50.2 (8.9)	behavioural intervention Plus DASH	behavioural intervention (includes goals for exercise, reducing sodium, alcohol, etc.)	dietary advice	Low
Lopes et al. 2003 - L	12	50	CO	USA	4	Y	OH, Lean	39 (6.9)	DASH ("DASH-CD" = DASH combination diet, high in antioxidants)	low antioxidant diet	dietary advice	Unclear
Lopes et al. 2003 - OB	12	50	CO	USA	4	Y	OB	35 (6.9)	DASH ("DASH-CD" = DASH combination diet, high in antioxidants)	low antioxidant diet	dietary advice	Unclear
Harsha et al. 2004	390	56	P	USA	4	Y	Hypertensive	48.5 (10.0)	DASH	typical American diet	metabolic	Unclear
Nowson et al. 2004	94	40	CO	Australia	4	N	Hypertensive	55.6 (9.9)	DASH-type diet (DASH plus increased fish, nuts, legumes, decreased sodium)	control diet (typical Australian diet)	dietary advice	Unclear

Nowson et al. 2005	54	0	P	Australia	12	Y	Hypertensive, OW (BMI 25-35kg/m2)	48.0 (9.3)	DASH-type diet (DASH plus increased fish, nuts, legumes, decreased sodium)	Low fat	dietary advice	Unclear
Nowson et al. 2009	95	100	P	Australia	14	Y	Hypertensive	59.2 (4.8)	DASH-type (DASH+low sodium, with lean meat)	Healthy diet - general guidelines to reduce fat, increase breads and cereals (represents a low fat diet)	dietary advice plus some supplementation of foods	Unclear
Al Solaiman et al. 2010 - L	15	80	CO	USA	3	N	Lean normotensive	36.7 (7.0)	DASH (without low fat dairy)	usual diet - low in fruit and vegetables	dietary advice	Unclear
Al Solaiman et al. 2010 - OB	15	80	CO	USA	3	N	OB, Prehypertensive + Hypertensive	40.3 (6.6)	DASH (without low fat dairy)	usual diet - low in fruit and vegetables	dietary advice	Unclear
Blumenthal et al. 2010	94	67	P	USA	16	Y	OW, Hypertensive, OH	51.8 (9.4)	DASH	usual diet	dietary advice	Low
Chen et al. 2010	290	49	P	USA	8	Y	Hypertensive (BP<160/80-95mmHg, not on antihypertensive meds, no poorly	~44 (11)	DASH	typical American diet	metabolic	Low

							controlled DM / dyslipidemia)					
Azadbakht et al. 2011	31	58	CO	Iran	8	Y	T2DM	55.0 (6.5)	DASH	usual diet	dietary advice	Low
Asemi et al. 2013	34	100	P	Iran	4	Y	GDM	30.1 (6.4)	DASH	usual GDM practice	dietary advice	Low

*For ROB, an assessment was performed using the Cochrane Risk of Bias tool, including the evaluation of individual domains of risk of bias (sequence generation, allocation concealment, blinding of participants/ personnel and outcome assessors, incomplete outcome data, selective outcome reporting). Each of the 5 domains was evaluated as either low, high or unclear ROB and the overall ROB category was determined based on the most selected category.

BMI, body mass index; BP, blood pressure; CO, crossover; DASH, dietary approaches to stop hypertension; DM, diabetes; F/U, follow-up; GDM, gestational diabetes; L, lean; LDL-C, low-density lipoprotein cholesterol; meds, medication; N, no; OB, obese; OH, overall healthy; OW, overweight; P, parallel; ROB, Risk of Bias; SD, standard deviation; T2DM, type 2 diabetes; Total-C, total-cholesterol; W, women; wks, weeks; Y, yes; yr; year

Table S18: Characteristics of controlled trials investigating the DASH dietary pattern and HDL-C

Study, yr	No. of participants	Sex (% F)	Design	Country	F/U, wks	Randomized	Metabolic Phenotype	Mean Age, yr (SD)	Intervention	Comparator	Feeding/Compliance	Overall ROB Category*
Appel et al. 2003	537	61	P	USA	24	Y	OH, Prehypertensive + Hypertensive	50.2 (8.9)	behavioural intervention Plus DASH	behavioural intervention (includes goals for exercise, reducing sodium, alcohol, etc.)	dietary advice	Low
Lopes et al. 2003 - L	12	50	CO	USA	4	Y	OH, Lean	39 (6.9)	DASH ("DASH-CD" = DASH combination diet, high in antioxidants)	low antioxidant diet	dietary advice	Unclear
Lopes et al. 2003 - OB	12	50	CO	USA	4	Y	OB	35 (6.9)	DASH ("DASH-CD" = DASH combination diet, high in antioxidants)	low antioxidant diet	dietary advice	Unclear
Harsha et al. 2004	390	56	P	USA	4	Y	Hypertensive	48.5 (10.0)	DASH	typical American diet	metabolic	Unclear
Nowson et al. 2004	94	40	CO	Australia	4	N	Hypertensive	55.6 (9.9)	DASH-type diet (DASH plus increased fish, nuts, legumes, decreased sodium)	control diet (typical Australian diet)	dietary advice	Unclear
Azadbakht et al. 2005 - M	22	0	P	Iran	24	Y	Metabolic Syndrome	~41.2 (12.3)	weight loss DASH	weight loss	dietary advice	Unclear

Azadbakht et al. 2005 - W	54	100	P	Iran	24	Y	Metabolic Syndrome	~41.2 (12.3)	weight loss DASH	weight loss	dietary advice	Unclear
Nowson et al. 2005	54	0	P	Australia	12	Y	Hypertensive, OW (BMI 25-35kg/m2)	48.0 (9.3)	DASH-type diet (DASH plus increased fish, nuts, legumes, decreased sodium)	Low fat	dietary advice	Unclear
Nowson et al. 2009	95	100	P	Australia	14	Y	Hypertensive	59.2 (4.8)	DASH-type (DASH+low sodium, with lean meat)	Healthy diet - general guidelines to reduce fat, increase breads and cereals (represents a low fat diet)	dietary advice plus some supplementation of foods	Unclear
Al Solaiman et al. 2010 - L	15	80	CO	USA	3	N	Lean normotensive	36.7 (7.0)	DASH (without low fat dairy)	usual diet - low in fruit and vegetables	dietary advice	Unclear
Al Solaiman et al. 2010 - OB	15	80	CO	USA	3	N	OB, Prehypertensive + Hypertensive	40.3 (6.6)	DASH (without low fat dairy)	usual diet - low in fruit and vegetables	dietary advice	Unclear
Blumenthal et al. 2010	94	67	P	USA	16	Y	OW, Hypertensive, OH	51.8 (9.4)	DASH	usual diet	dietary advice	Low

Chen et al. 2010	290	49	P	USA	8	Y	Hypertensive (BP<160/80-95mmHg, not on antihypertensive meds, no poorly controlled DM /dyslipidemia)	~44 (11)	DASH	typical American diet	metabolic	Low
Azadbakht et al. 2011	31	58	CO	Iran	8	Y	T2DM	55.0 (6.5)	DASH	usual diet	dietary advice	Low
Asemi et al. 2013	34	100	P	Iran	4	Y	GDM	30.1 (6.4)	DASH	usual GDM practice	dietary advice	Low

*For ROB, an assessment was performed using the Cochrane Risk of Bias tool, including the evaluation of individual domains of risk of bias (sequence generation, allocation concealment, blinding of participants/ personnel and outcome assessors, incomplete outcome data, selective outcome reporting). Each of the 5 domains was evaluated as either low, high or unclear ROB and the overall ROB category was determined based on the most selected category.

BMI, body mass index; BP, blood pressure; CO, crossover; DASH, dietary approaches to stop hypertension; DM, diabetes; F, female; F/U, follow-up; GDM, gestational diabetes; HDL-C, high-density lipoprotein cholesterol; L, lean; M, male; meds, medication; N, no; OB, obese; OH, overall healthy; OW, overweight; P, parallel; ROB, Risk of Bias; SD, standard deviation; T2DM, type 2 diabetes; W, women; wks, weeks; Y, yes; yr; year

Table S19: Characteristics of controlled trials investigating the DASH dietary pattern and triglycerides

Study, yr	No. of participants	Sex (% F)	Design	Country	F/U, wks	Randomized	Metabolic Phenotype	Mean Age, yr (SD)	Intervention	Comparator	Feeding/Compliance	Overall ROB Category*
Appel et al. 2003	537	61	P	USA	24	Y	OH, Prehypertensive + hypertensive	50.2 (8.9)	behavioural intervention Plus DASH	behavioural intervention (includes goals for exercise, reducing sodium, alcohol, etc.)	dietary advice	Low
Lopes et al. 2003 - L	12	50	CO	USA	4	Y	OH, lean	39 (6.9)	DASH ("DASH-CD" = DASH combination diet, high in antioxidants)	low antioxidant diet	dietary advice	Unclear
Lopes et al. 2003 - OB	12	50	CO	USA	4	Y	OB	35 (6.9)	DASH ("DASH-CD" = DASH combination diet, high in antioxidants)	low antioxidant diet	dietary advice	Unclear
Harsha et al. 2004	390	56	P	USA	4	Y	Hypertensive	48.5 (10.0)	DASH	typical American diet	metabolic	Unclear
Nowson et al. 2004	94	40	CO	Australia	4	N	Hypertensive	55.6 (9.9)	DASH-type diet (DASH plus increased fish, nuts, legumes, decreased sodium)	control diet (typical Australian diet)	dietary advice	Unclear

Azadbakht et al. 2005 - M	22	0	P	Iran	24	Y	Metabolic Syndrome	~41.2 (12.3)	weight loss DASH	weight loss	dietary advice	Unclear
Azadbakht et al. 2005 - W	54	100	P	Iran	24	Y	Metabolic Syndrome	~41.2 (12.3)	weight loss DASH	weight loss	dietary advice	Unclear
Nowson et al. 2005	54	0	P	Australia	12	Y	Hypertensive, OW (BMI 25-35kg/m2)	48.0 (9.3)	DASH-type diet (DASH plus increased fish, nuts, legumes, decreased sodium)	Low fat	dietary advice	Unclear
Al Solaiman et al. 2010 - L	15	80	CO	USA	3	N	Lean normotensive	36.7 (7.0)	DASH (without low fat dairy)	usual diet - low in fruit and vegetables	dietary advice	Unclear
Al Solaiman et al. 2010 - OB	15	80	CO	USA	3	N	OB, Prehypertensive + Hypertensive	40.3 (6.6)	DASH (without low fat dairy)	usual diet - low in fruit and vegetables	dietary advice	Unclear
Blumenthal et al. 2010	94	67	P	USA	16	Y	OW, Hypertensive, OH	51.8 (9.4)	DASH	usual diet	dietary advice	Low

Chen et al. 2010	290	49	P	USA	8	Y	Hypertensive (BP<160/80-95mmHg, not on antihypertensive meds, no poorly controlled DM / dyslipidemia)	~44 (11)	DASH	typical American diet	metabolic	Low
Azadbakht et al. 2011	31	58	CO	Iran	8	Y	T2DM	55.0 (6.5)	DASH	usual diet	dietary advice	Low
Asemi et al. 2013	34	100	P	Iran	4	Y	GDM	30.1 (6.4)	DASH	usual GDM practice	dietary advice	Low

*For ROB, an assessment was performed using the Cochrane Risk of Bias tool, including the evaluation of individual domains of risk of bias (sequence generation, allocation concealment, blinding of participants/ personnel and outcome assessors, incomplete outcome data, selective outcome reporting). Each of the 5 domains was evaluated as either low, high or unclear ROB and the overall ROB category was determined based on the most selected category.

BMI, body mass index; BP, blood pressure; CO, crossover; DASH, dietary approaches to stop hypertension; DM, diabetes; F, female; F/U, follow-up; GDM, gestational diabetes; L, lean; M, male; meds, medication; N, no; OB, obese; OH, overall healthy; OW, overweight; P, parallel; ROB, Risk of Bias; SD, standard deviation; T2DM, type 2 diabetes; W, women; wks, weeks; Y, yes; yr; year

Table S20: Search strategy for controlled clinical trials assessing the effect of the DASH diet intervention on HbA1c

Database	Search Period	Search Terms
Medline	1946 to November 27, 2018	1 DASH.mp<br>2 dietary approaches to stop hypertension.mp<br>3 dietary pattern.mp<br>4 1 OR 2 OR 3<br>5 glyc?em*.mp<br>6 exp insulin/<br>7 HbA1c.mp<br>8 A1c.mp<br>9 hemoglobin A, glycosylated.mp<br>10 fructosamine.mp<br>11 exp blood glucose/<br>12 gly*albumin.mp<br>13 5 OR 6 OR 7 OR 8 OR 9 OR 10 OR 11 OR 12<br>14 4 AND 13<br>15 limit 14 to animals<br>16 14 NOT 15
Embase	1946 to November 27, 2018	1 DASH.mp<br>2 dietary approaches to stop hypertension.mp<br>3 dietary pattern.mp<br>4 1 OR 2 OR 3<br>5 glyc?em*.mp<br>6 exp insulin/<br>7 HbA1c.mp<br>8 A1c.mp<br>9 hemoglobin A, glycosylated.mp<br>10 fructosamine.mp<br>11 exp blood glucose/<br>12 gly*albumin.mp<br>13 5 OR 6 OR 7 OR 8 OR 9 OR 10 OR 11 OR 12<br>14 4 AND 13<br>15 limit 14 to animals<br>16 14 NOT 15

The Cochrane Library	1946 to November 27, 2018	1	DASH.mp
		2	dietary approaches to stop hypertension.mp
		3	dietary pattern.mp
		4	1 OR 2 OR 3
		5	glyc?em*.mp
		6	exp insulin/
		7	HbA1c.mp
		8	A1c.mp
		9	hemoglobin A, glycosylated.mp
		10	fructosamine.mp
		11	exp blood glucose/
		12	gly*albumin.mp
		13	5 OR 6 OR 7 OR 8 OR 9 OR 10 OR 11 OR 12
		14	4 AND 13
		15	limit 14 Medline and EMBASE

Table S21: Characteristics of controlled trials investigating the DASH dietary pattern and HbA1c

Study, yr	No. of participants	Sex (% F)	Design	Country	F/U, wks	Randomized	Metabolic Phenotype	Mean Age, yr (SD)	Intervention	Comparator	Feeding/Compliance	Overall ROB Category*
Azadbakht et al. 2011	31	58	CO	Iran	8	Y	T2DM	55.0 (6.5)	DASH	usual diet	dietary advice	Unclear
Asemi et al. 2013	34	100	P	Iran	4	Y	GDM	30.1 (6.4)	DASH	usual GDM practice	dietary advice	Low

Table S22: Characteristics of controlled trials investigating the DASH dietary pattern and blood glucose

Study, yr	No. of participants	Sex (% F)	Design	Country	F/U, wks	Randomized	Metabolic Phenotype	Mean Age, yr (SD)	Intervention	Comparator	Feeding/Compliance	Overall ROB Category*
Appel et al. 2003	537	61	P	USA	24	Y	OH, Prehypertensive + Hypertensive	50.2 (8.9)	behavioural intervention Plus DASH	behavioural intervention (includes goals for exercise, reducing sodium, alcohol, etc.)	dietary advice	Low
Lopes et al. 2003 - L	12	50	CO	USA	4	Y	OH, Lean	39 (6.9)	DASH ("DASH-CD" = DASH combination diet, high in antioxidants)	low antioxidant diet	dietary advice	Unclear
Lopes et al. 2003 - OB	12	50	CO	USA	4	Y	OB	35 (6.9)	DASH ("DASH-CD" = DASH combination diet, high in antioxidants)	low antioxidant diet	dietary advice	Unclear
Azadbakht et al. 2005 - M	22	0	P	Iran	24	Y	Metabolic Syndrome	~41.2 (12.3)	weight loss DASH	weight loss	dietary advice	Unclear
Azadbakht et al. 2005 - W	54	100	P	Iran	24	Y	Metabolic Syndrome	~41.2 (12.3)	weight loss DASH	weight loss	dietary advice	Unclear

Blumenthal et al. 2010	94	67	P	USA	16	Y	OW, Hypertensive, OH	51.8 (9.4)	DASH	usual diet	dietary advice	Low
Al Solaiman et al. 2010 - L	15	80	CO	USA	3	N	Lean normotensive	36.7 (7.0)	DASH (without low fat dairy)	usual diet - low in fruit and vegetables	dietary advice	Unclear
Al Solaiman et al. 2010 - OB	15	80	CO	USA	3	N	OB, Prehypertensive + Hypertensive	40.3 (6.6)	DASH (without low fat dairy)	usual diet - low in fruit and vegetables	dietary advice	Unclear
Azadbakht et al. 2011	31	58	CO	Iran	8	Y	T2DM	55.0 (6.5)	DASH	usual diet	dietary advice	Low
Asemi et al. 2013	34	100	P	Iran	4	Y	GDM	30.1 (6.4)	DASH	usual GDM practice	dietary advice	Low

*For ROB, an assessment was performed using the Cochrane Risk of Bias tool, including the evaluation of individual domains of risk of bias (sequence generation, allocation concealment, blinding of participants/ personnel and outcome assessors, incomplete outcome data, selective outcome reporting). Each of the 5 domains was evaluated as either low, high or unclear ROB and the overall ROB category was determined based on the most selected category.

CO, crossover; DASH, dietary approaches to stop hypertension; F, female; F/U, follow-up; GDM, gestational diabetes; L, lean; M, male; N, no; OB, obese; OH, overall healthy; OW, overweight; P, parallel; ROB, Risk of Bias; SD, standard deviation; T2DM, type 2 diabetes; W, women; wks, weeks; Y, yes; yr; year

Table S23: Characteristics of controlled trials investigating the DASH dietary pattern and fasting insulin

Study, yr	No. of participants	Sex (% F)	Design	Country	F/U, wks	Randomized	Metabolic Phenotype	Mean Age, yr (SD)	Intervention	Comparator	Feeding /Compliance	Overall ROB Category*
Lopes et al. 2003 - L	12	50	CO	USA	4	Y	OH, Lean	39 (6.9)	DASH ("DASH-CD" = DASH combination diet, high in antioxidants)	low antioxidant diet	dietary advice	Unclear
Ard et al. 2004	36	67	P	USA	24	Y	OH, Prehypertensive + Hypertensive	51.8	behavioural intervention Plus DASH	advice only	dietary advice	Low
Lien et al. 2007 - OH	265	66	P	USA	24	Y	OH, Prehypertensive + Hypertensive	~49.8 (9.1)	behavioural intervention Plus DASH	advice only	dietary advice	Low
Al Solaiman et al. 2009 - salt sensitive	9	78	CO	USA	3	N	OH	44.1 (1.4)	DASH (without low fat dairy)	usual diet - low in fruit and vegetables	dietary advice	Unclear
Al Solaiman et al.	9	78	CO	USA	3	N	OW, Hypertensive, OH	34.3 (2.5)	DASH (without low fat dairy)	usual diet - low in fruit and vegetables	dietary advice	Unclear

2009 - salt resistant												
Hodson et al. 2010	27	41	P	UK	4	N	OH, OW	~45.5	DASH intermediate sodium	habitual diet/lifestyle	dietary advice, DASH group supplemented with no salt sunflower spread and olive oil	Unclear
Blumenthal et al. 2010	94	67	P	USA	16	Y	OW, Hypertensive, OH	51.8 (9.4)	DASH	usual diet	dietary advice	Low
Al Solaiman et al. 2010 - L	15	80	CO	USA	3	N	Lean normotensive	36.7 (7.0)	DASH (without low fat dairy)	usual diet - low in fruit and vegetables	dietary advice	Unclear
Lopes et al. 2003 - OB	12	50	CO	USA	4	Y	OB	35 (6.9)	DASH ("DASH-CD" = DASH combination diet, high in antioxidants)	low antioxidant diet	dietary advice	Unclear
Lien et al. 2007 -	266	58	P	USA	24	Y	OH, Prehypertensive +	~49.8 (9.1)	behavioural intervention Plus DASH	advice only	dietary advice	Low

MetS/ DysL							Hypertensive					
Al Solaiman et al. 2010 - OB	15	80	CO	USA	3	N	OB, Prehypertensive + Hypertensive	40.3 (6.6)	DASH (without low fat dairy)	usual diet - low in fruit and vegetables	dietary advice	Unclear

*For ROB, an assessment was performed using the Cochrane Risk of Bias tool, including the evaluation of individual domains of risk of bias (sequence generation, allocation concealment, blinding of participants/ personnel and outcome assessors, incomplete outcome data, selective outcome reporting). Each of the 5 domains was evaluated as either low, high or unclear ROB and the overall ROB category was determined based on the most selected category.

CO, crossover; DASH, dietary approaches to stop hypertension; DysL, dyslipidemia; F, female; F/U, follow-up; L, lean; MetS, metabolic syndrome; N, no; OB, obese; OH, overall healthy; OW, overweight; P, parallel; ROB, Risk of Bias; SD, standard deviation; wks, weeks; Y, yes; yr; year

Table S24: Characteristics of controlled trials investigating the DASH dietary pattern and HOMA-IR

Study, yr	No. of participants	Sex (% F)	Design	Country	F/U, wks	Randomized	Metabolic Phenotype	Mean Age, yr (SD)	Intervention	Comparator	Feeding /Compliance	Overall ROB Category*
Lopes et al. 2003 - L	12	50	CO	USA	4	Y	OH, Lean	39 (6.9)	DASH ("DASH-CD" = DASH combination diet, high in antioxidants)	low antioxidant diet	dietary advice	Unclear
Lopes et al. 2003 - OB	12	50	CO	USA	4	Y	OB	35 (6.9)	DASH ("DASH-CD" = DASH combination diet, high in antioxidants)	low antioxidant diet	dietary advice	Unclear
Lien et al. 2007 - OH	265	66	P	USA	24	Y	OH, Prehypertensive + Hypertensive	~49.8 (9.1)	behavioural intervention Plus DASH	advice only	dietary advice	Low
Lien et al. 2007 - MetS/ DysL	266	58	P	USA	24	Y	OH, Prehypertensive + Hypertensive	~49.8 (9.1)	behavioural intervention Plus DASH	advice only	dietary advice	Low
Al Solaiman et al. 2009 - salt sensitive	9	78	CO	USA	3	N	OH	44.1 (1.4)	DASH (without low fat dairy)	usual diet - low in fruit and vegetables	dietary advice	Unclear

Al Solaiman et al. 2009 - salt resistant	9	78	CO	USA	3	N	OW, Hypertensive, OH	34.3 (2.5)	DASH (without low fat dairy)	usual diet - low in fruit and vegetables	dietary advice	Unclear
Al Solaiman et al. 2010 - L	15	80	CO	USA	3	N	Lean normotensive	36.7 (7.0)	DASH (without low fat dairy)	usual diet - low in fruit and vegetables	dietary advice	Unclear
Al Solaiman et al. 2010 - OB	15	80	CO	USA	3	N	OB, Prehypertensive + Hypertensive	40.3 (6.6)	DASH (without low fat dairy)	usual diet - low in fruit and vegetables	dietary advice	Unclear

*For ROB, an assessment was performed using the Cochrane Risk of Bias tool, including the evaluation of individual domains of risk of bias (sequence generation, allocation concealment, blinding of participants/ personnel and outcome assessors, incomplete outcome data, selective outcome reporting). Each of the 5 domains was evaluated as either low, high or unclear ROB and the overall ROB category was determined based on the most selected category.

CO, crossover; DASH, dietary approaches to stop hypertension; DysL, dyslipidemia; F, female; F/U, follow-up; HOMA-IR, Homeostatic Model Assessment of Insulin Resistance; L, lean; MetS, metabolic syndrome; N, no; OB, obese; OH, overall healthy; OW, overweight; P, parallel; ROB, Risk of Bias; SD, standard deviation; wks, weeks; Y, yes; yr; year

Table S25: Characteristics of controlled trials investigating the DASH dietary pattern and body weight

Study, yr	No. of participants	Sex (% F)	Design	Country	F/U, wks	Randomized	Metabolic Phenotype	Mean Age, yr (SD)	Intervention	Comparator	Feeding /Compliance	Overall ROB Category*
Ard et al. 2004	53	71	P	USA	52	Y	Hypertensive (BP<160/ 80-95mmHg, not on antihypertensive meds, no poorly controlled DM /dyslipidemia)	~49.02 (10.73)	DASH	typical American diet	dietary advice	Unclear
Nowson et al. 2005	54	0	P	Australia	12	Y	Hypertensive, OW (BMI 25-35kg/m2)	48.0 (9.3)	DASH-type diet (DASH plus increased fish, nuts, legumes, decreased sodium)	Low fat	dietary advice	Unclear
Elmer et al. 2006	476	~61.1	P	USA	24	Y	OH, Prehypertensive + Hypertensive	~50.2 (8.9)	behavioural intervention Plus DASH	behavioural intervention (includes goals for exercise, reducing sodium, alcohol, etc.)	dietary advice	Low
Nowson et al. 2009	95	100	P	Australia	14	Y	Hypertensive	59.2 (4.8)	DASH-type (DASH+low sodium, with lean meat)	Healthy diet - general guidelines to reduce fat, increase breads and cereals (represents a	dietary advice plus some	Unclear

										low fat diet)	supplementation of foods	
Blumenthal et al. 2010	95	66	P	USA	16	Y	OW, Hypertensive, OH	51.8 (9.4)	DASH	usual diet	dietary advice	Low
Lima et al. 2013	206	78	P	Brazil	24	Y	Hypertensive	NR	DASH-Na + LGI	low sodium standard HTN advice	dietary advice	Low
Rifai et al. 2015	48	40	P	USA	12	Y	Heart Failure patients	62.0 (11.6)	DASH	general HF recommendations	dietary advice	Unclear
Azadbakht et al. 2005 - M	22	0	P	Iran	24	Y	Metabolic Syndrome	~41.2 (12.3)	weight loss DASH	weight loss	dietary advice	Unclear
Azadbakht et al. 2005 - W	54	100	P	Iran	24	Y	Metabolic Syndrome	~41.2 (12.3)	weight loss DASH	weight loss	dietary advice	Unclear
Asemi et al. 2014	48	100	P	Iran	8	Y	OW/OB, PCOS	30.1 (6.4)	weight loss DASH (F/V, LF dairy, red Na, SF, chol, refined grains/sweets)	weight loss traditional Iranian diet	dietary advice	Low
Razavi Zade et al.	60	50	P	Iran	8	Y	OW/OB, NAFLD	41.3 (9.2)	weight loss DASH (F/V, LF dairy, red Na, SF,	weight loss traditional Iranian diet	dietary advice	Low

2015									chol, refined grains/sweets)			

*For ROB, an assessment was performed using the Cochrane Risk of Bias tool, including the evaluation of individual domains of risk of bias (sequence generation, allocation concealment, blinding of participants/ personnel and outcome assessors, incomplete outcome data, selective outcome reporting). Each of the 5 domains was evaluated as either low, high or unclear ROB and the overall ROB category was determined based on the most selected category.

BMI, body mass index; BP, blood pressure; chol, cholesterol; DASH, dietary approaches to stop hypertension; DM, diabetes; F, female; F/U, follow-up; F/V, fruit and vegetable; HF, heart failure; HTN, hypertension; LF, low fat; LGI, low glycemic index; M, male; meds, medication; Na, sodium; NAFLD, non-alcoholic fatty liver disease; NR, not reported; OB, obese; OH, overall healthy; OW, overweight; P, parallel; PCOS, polycystic ovarian syndrome; red, reduced; ROB, Risk of Bias; SD, standard deviation; SF, saturated fat; W, women; wks, weeks; Y, yes; yr; year

Table S26: Characteristics of controlled trials investigating the DASH dietary pattern and CRP

Study, yr	No. of participants	Sex (% F)	Design	Country	F/U, wks	Randomized	Metabolic Phenotype	Mean Age, yr (SD)	Intervention	Comparator	Feeding /Compliance	Overall ROB Category*
King et al. 2007	35	80	CO	USA	3	Y	Lean normotensive and OB hypertensive	38.3 (1.2)	DASH high fibre	usual diet supplemented with 30g/d psyllium, potassium and magnesium to match DASH	dietary advice	Unclear
Roussell et al. 2012	36	58	CO	USA	5	Y	OH, Hyperlipidemia	50.0 (8.4)	DASH	healthy American diet (higher in fat, lower in fibre)	metabolic	Unclear
Jenkins et al. 2015	241	61	P	Canada	24	Y	Hyperlipidemic	20-85	DASH-type lacto-ovo vegetarian	Portfolio diet (plant-based) with soy protein, viscous fibers and nuts	dietary advice	Low
Azadbakht et al. 2011	31	58	CO	Iran	8	Y	T2DM	55.0 (6.5)	DASH	usual diet	dietary advice	Low
Asemi et al. 2014	48	100	P	Iran	8	Y	OW/OB, PCOS	30.1 (6.4)	weight loss DASH (F/V, LF dairy, red Na, SF, chol, refined grains/sweets)	weight loss traditional Iranian diet	dietary advice	Low

Razavi Zade et al. 2015	60	50	P	Iran	8	Y	OW/OB, NAFLD	41.3 (9.2)	weight loss DASH (F/V, LF dairy, red Na, SF, chol, refined grains/sweets)	weight loss traditional Iranian diet	dietary advice	Low

*For ROB, an assessment was performed using the Cochrane Risk of Bias tool, including the evaluation of individual domains of risk of bias (sequence generation, allocation concealment, blinding of participants/ personnel and outcome assessors, incomplete outcome data, selective outcome reporting). Each of the 5 domains was evaluated as either low, high or unclear ROB and the overall ROB category was determined based on the most selected category.

chol, cholesterol; CO, crossover; CRP, c-reactive protein; DASH, dietary approaches to stop hypertension; F, female; F/U, follow-up; F/V, fruit and vegetable; HF, heart failure; HTN, hypertension; LF, low fat; Na, sodium; NAFLD, non-alcoholic fatty liver disease; OB, obese; OH, overall healthy; OW, overweight; P, parallel; PCOS, polycystic ovarian syndrome; red, reduced; ROB, Risk of Bias; SD, standard deviation; SF, saturated fat; wks, weeks; Y, yes; yr; year

Figure S1: Risk of Bias summary for all controlled trials included in the systematic reviews and meta-analyses of cardiovascular risk factors

	Random Sequence Generation	Allocation Concealment	Blinding of Participants, Personnel and Outcome Assessment	Incomplete Outcome Data	Selective Reporting
Al Solaiman et al. 2009	?	?	−	−	+
Al Solamain et al. 2010	?	?	−	?	+
Appel et al. 1997	?	?	+	+	+
Appel et al. 2003	+	+	+	+	+
Ard et al. 2004 -Diabetes Care	?	?	+	?	+
Ard et al. 2004 - Am J Hypertens	+	+	?	+	+
Asemi et al. 2013	+	?	+	?	+
Asemi et al. 2014*	+	?	+	?	+
Azadbakht et al. 2005	+	?	?	+	?
Azadbakht et al. 2011	+	?	+	?	+
Blumenthal et al. 2010	+	+	+	+	+
Chen et al. 2010	?	?	+	+	+
Conlin et al. 2003	?	?	+	+	+
Edwards et al. 2011	+	?	?	?	?
Elmer et al. 2006	+	+	?	?	+
Harsha et al. 2004	?	?	+	?	+

	Random Sequence Generation	Allocation Concealment	Blinding of Participants, Personnel and Outcome Assessment	Incomplete Outcome Data	Selective Reporting
Hodson et al. 2010	−	−	?	+	+
Jenkins et al. 2015	+	+	+	+	+
King et al. 2007	?	?	?	?	?
Lien et al. 2007	+	+	?	+	+
Lima et al. 2013	+	+	?	?	+
Lin et al. 2012	+	?	+	+	+
Lopes et al. 2003	?	?	?	+	?
Malloy-McFall et al. 2010	+	+	?	+	?
Nowson et al. 2004	+	−	?	+	?
Nowson et al. 2005	+	−	?	+	?
Nowson et al. 2009	?	?	?	?	?
Razavi Zade et al. 2015	+	?	+	+	+
Rifai et al. 2015	+	?	−	?	+
Roussell et al. 2012	?	?	?	?	+
Sacks et al. 2001	?	?	+	+	+

*the reference of this study as reported in Soltani et al. 2016 was incorrectly recorded as published in 2015

Green dots indicate low risk of bias, red dots indicate high risk of bias and yellow dots indicate unclear risk of bias.

Figure S2: Risk of Bias graph for all controlled trials included in the systematic reviews and meta-analyses of cardiovascular risk factors

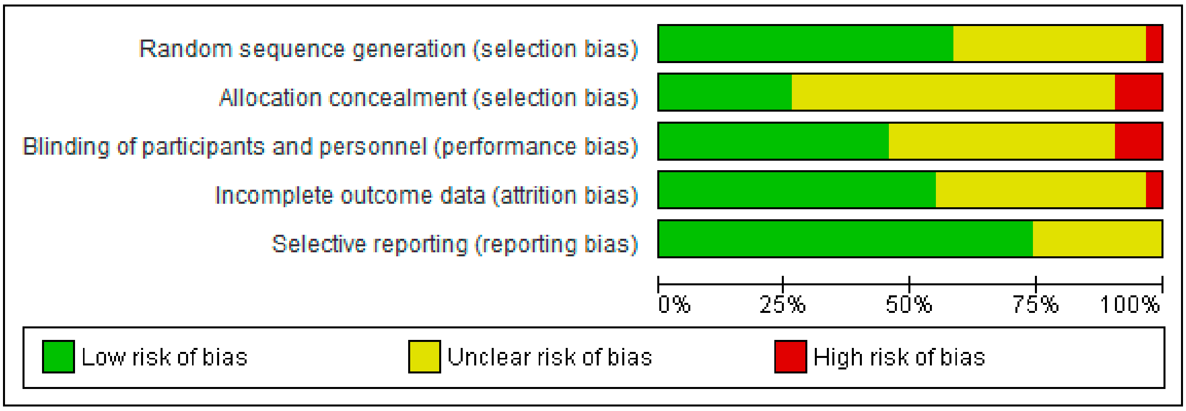

Colored bars represent the proportion of studies assessed as low (green), unclear (yellow) or high (red) risk of bias for the 5 domains of bias above according to criteria set by the Cochrane Risk of Bias tool in the 31 included unique trials.

Figure S3: Literature search for the effect of the DASH diet intervention on HbA1c in controlled clinical trials

All reports identified through database searching: 879
(through 27 November, 2018)
MEDLINE: 269
EMBASE: 610
The Cochrane Library: 0
Manual Searches: 0

↓

Total reports after duplicates removed: 629

→ **Reports excluded by title and abstract: 597**
Observational: 341
Review: 145
Letter/Editorial: 3
Protocol: 6
Drug: 4
In vitro: 2
Non-human: 1
No control group: 7
No assessment of DASH diet intervention: 85
Co-intervention: 2
Companion paper: 1

↓

Reports assessed for full review: 32

→ **Reports excluded by full review: 30**
Observational: 0
Review: 0
Letter/Editorial: 1
Protocol: 0
Drug: 0
In vitro: 0
Non-human: 0
No control group: 2
No assessment of DASH diet intervention: 1
Co-intervention: 2
Companion paper: 4
No viable outcome data: 20

↓

Total reports included in meta-analysis: 2

Figure S4: Forest plot for controlled trials investigating the effect of the DASH dietary pattern on HbA1c.

Study	N DASH	N Control	Weight (%)	Mean difference (95% CI) HbA1c (%)
Asemi et al. 2013	17	17	70.9	-0.25 [-0.136, -0.14]
Azadbakht et al. 2011	31	31	29.1	-1.20 [-1.38, -1.02]
**Total**				**-0.53 [-0.62, -0.43]**

Heterogeneity: $Chi^2 = 77.15$, $df = 1$ ($P < 0.00001$); $I^2 = 99\%$

Test for overall effect: $Z = 10.71$ ($P < 0.00001$)

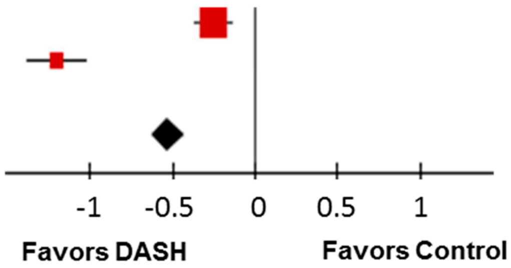

Forest plot for controlled trials investigating the effect of the DASH dietary patterns on HbA1c. The overall effect estimate is represented by the diamond. Data are expressed as weighted mean differences with 95% confidence intervals (CIs), using the generic inverse-variance method with the fixed effects model. Paired analyses were applied to all crossover studies. Inter-study heterogeneity was tested by the Cochran Q-statistic at a significance level of $p < 0.10$ and quantified by I2, level of ≥ 50 % represented substantial heterogeneity.

DASH, dietary approaches to stop hypertension; HbA1c, hemoglobin A1c.
